# Biomimetic dual-catalytic nanozyme remodels the liver fibrotic microenvironment to alleviate fibrosis

**DOI:** 10.1093/rb/rbag163

**Published:** 2026-11-03

**Authors:** Xueyao Tang, Juying Zhang, Jixuan Zhang, Lei Huang, Changbao Li, Hong Zhou, Ming Ye, Jujie Du, Yang Liu, Yang Zhou, Ying Liu

**Affiliations:** Department of Ultrasound, Affiliated Hospital of Southwest Jiaotong University, The Third People’s Hospital of Chengdu, Chengdu 610031, China; School of Medical Imaging, North Sichuan Medical College, Nanchong 637000, China; School of Medical Imaging, North Sichuan Medical College, Nanchong 637000, China; College of Medicine, Southwest Jiaotong University, Chengdu 610031, China; Department of Ultrasound, Huili People’s Hospital, Liangshan 615100, China; Department of Ultrasound, Affiliated Hospital of Southwest Jiaotong University, The Third People’s Hospital of Chengdu, Chengdu 610031, China; Department of Ultrasound, Affiliated Hospital of Southwest Jiaotong University, The Third People’s Hospital of Chengdu, Chengdu 610031, China; Department of Ultrasound, Affiliated Hospital of Southwest Jiaotong University, The Third People’s Hospital of Chengdu, Chengdu 610031, China; Department of Ultrasound, Affiliated Hospital of Southwest Jiaotong University, The Third People’s Hospital of Chengdu, Chengdu 610031, China; Department of Ultrasound, Affiliated Hospital of Southwest Jiaotong University, The Third People’s Hospital of Chengdu, Chengdu 610031, China; Department of Ultrasound, Affiliated Hospital of Southwest Jiaotong University, The Third People’s Hospital of Chengdu, Chengdu 610031, China

**Keywords:** nanozyme, liver fibrosis, hepatic stellate cell-targeted therapy, ROS scavenging, manganese dioxide

## Abstract

Activated hepatic stellate cells (HSCs) are the primary drivers of pathological extracellular matrix accumulation in liver fibrosis. Thus, inhibiting HSC activation is crucial for treatment; however, therapeutic efficacy often falls short of expectations. A major obstacle is the hypoxic, reactive oxygen species (ROS)-rich microenvironment, which fuels persistent HSC activation and compromises long-term treatment success. Here, we developed an activated HSC-targeted biomimetic nanozyme, HSC-HMMDN, by coating hollow mesoporous manganese dioxide nanoparticles (HMMDN) with membranes derived from activated HSCs to synergistically clear ROS and alleviate hypoxia. This homologous coating strategy enables precise recognition of surface adhesion molecules (such as integrins and N-cadherin) that are highly expressed on activated HSCs, ensuring targeted delivery. Once there, the nanozyme, endowed with superoxide dismutase- and catalase-like activities, scavenges excessive ROS, revives mitochondrial function and suppresses the TGF-β/Smad cascade to inhibit HSC activation. In a carbon tetrachloride (CCl_4_)-induced liver fibrosis rat model, HSC-HMMDN significantly ameliorated fibrosis and alleviated the hypoxic microenvironment without showing detectable toxicity. Overall, this targeted microenvironment remodeling strategy provides a new avenue for liver fibrosis therapy.

## Graphical abstract

**Graphical Abstract:**
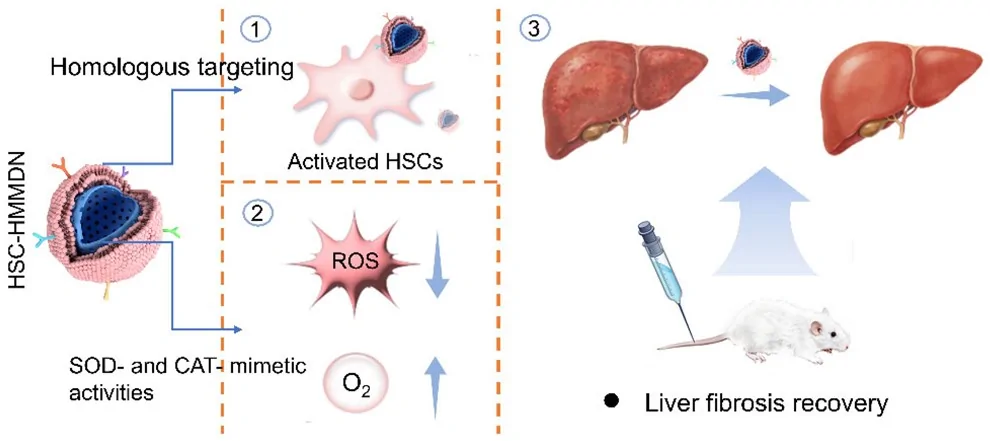

## Introduction

Liver fibrosis is a dynamic tissue repair resulting from repeated injury to the liver parenchyma, characterized by extensive extracellular matrix (ECM) deposition [1, 2]. Annually, 2% to 3% of patients advance to cirrhosis or hepatocellular carcinoma, leading to 150 000–300 000 deaths and constituting approximately 45% of worldwide liver disease mortality [3]. Despite considerable investment of manpower and material resources in potential therapies, success in treating liver fibrosis has remained elusive, largely due to the complexity of its underlying pathological mechanisms [4, 5]. Consequently, to improve clinical outcomes, it is imperative to gain deeper insights into the disease mechanisms and develop more reliable therapies [6].

Activated hepatic stellate cells (HSCs) are regarded as the central drivers of liver fibrosis, functioning as the principal source of ECM deposition [7, 8]. It is well recognized that HSCs are located predominantly in the space of Disse, constituting only 5–8% of total liver cells [9]. Upon chronic liver injury, however, quiescent HSCs become activated and proliferate, exhibiting myofibroblast characteristics and secreting large amounts of ECM, which ultimately leads to the disruption and remodeling of the liver tissue architecture [10, 11]. Multiple complicated signaling pathways, in which the transforming growth factor-β (TGF-β)/Smad signaling pathway plays a crucial role, regulate HSC activation [12]. Through the phosphorylation of key downstream effectors, notably Smad2/3, TGF-β activates these Smad proteins, enabling their nuclear translocation and driving the expression of fibrosis-related genes such as α-smooth muscle actin (α-SMA) and collagen-I [9, 13–15]. Therefore, inhibiting the TGF-β/Smad signaling in HSCs has emerged as a promising therapeutic strategy to suppress their activation and, consequently, counteract liver fibrosis [14, 16–18]. However, the main obstacle to achieving persistent therapeutic efficacy primarily resides in the unique pathological microenvironment of fibrotic lesions, characterized by the coexistence of hypoxia and excessive reactive oxygen species (ROS), which continuously drives the reactivation of fibrogenic pathways.

Tissue hypoxia caused by impaired microcirculation initiates a self-amplifying fibrogenic loop. Under hypoxic conditions, hypoxia-inducible factor-1α (HIF-1α) is stabilized [19]. In addition to directly promoting HSC activation, HIF-1α upregulates pro-oxidant enzymes [e.g. NADPH oxidase 4 (NOX4)] and disrupts mitochondrial function, triggering a surge of ROS, particularly hydrogen peroxide (H_2_O_2_), in activated HSCs [20–23]. Critically, the overproduction of ROS serves as a direct intracellular signaling molecule that continuously reactivates downstream pathways such as TGF-β/Smad, thereby driving HSC activation and the progression of fibrosis [18, 24–26]. The resulting ECM accumulation further worsens microcirculatory impairment and hypoxia, reinforcing the HIF-1α/ROS axis and establishing a vicious cycle. Therefore, effectively disrupting this loop likely requires simultaneously reducing ROS and relieving hypoxia, thereby suppressing upstream drivers of fibrogenesis, including TGF-β/Smad signaling.

Recent years have proved the rapid development of nanozyme systems with enzyme-mimicking activities for diverse biomedical applications [27–31]. Among these, manganese dioxide (MnO_2_) is a promising material for liver fibrosis therapy because of its variable valence chemistry, favorable biosafety, MRI capability, structural tunability and ease of functionalization [32–34]. Specifically, by leveraging the variable valence states of manganese, MnO_2_ mimics natural superoxide dismutase (SOD) and catalase (CAT), allowing it to scavenge superoxide anions (O_2_•^-^) and decompose H_2_O_2_ [35–37]. More importantly, these reactions can generate O_2_  *in situ*, directly alleviating tissue hypoxia and disrupting the HIF-1α/ROS loop at its source. Notably, the therapeutic efficacy of MnO_2_ can be further amplified by rational structural design. A hollow mesoporous architecture, for instance, provides a higher specific surface area and more active sites, which collectively maximize catalytic efficiency and offer potential for drug loading. Therefore, hollow mesoporous MnO_2_ nanoparticles (HMMDN) constitute a remarkable nanozyme platform for liver fibrosis treatment, synergizing strong antioxidant capability with advantageous structural features. Unfortunately, owing to the dense ECM and capillarized sinusoids in the fibrotic liver, unconjugated HMMDN is unlikely to reach sufficient concentrations in activated HSCs, presenting a formidable barrier to drug delivery.

Although antibody-based targeting can improve specificity for activated HSCs [38, 39], it is often limited by complex fabrication, limited *in vivo* stability and potential safety concerns. To address this, biomimetic modifications have attracted considerable attention [40, 41]. By preserving native membrane proteins, cell-membrane coatings can confer ‘self-identification’ cues that promote homologous targeting and enhance accumulation at fibrotic lesions while reducing off-target interactions [42]. In particular, this biological ‘camouflage’ facilitates immune evasion and prolongs systemic circulation, thereby improving delivery efficiency [43].

Building upon this rationale, we engineered a biomimetic nano-delivery system, termed HSC-HMMDN, by coating HMMDN with membranes derived from activated HSCs to enable targeted microenvironment remodeling for liver fibrosis therapy (Figure 1). We anticipate that HSC-HMMDN effectively targets fibrotic liver tissue and is internalized by activated HSCs via homologous membrane recognition. After cellular uptake, HMMDN should leverage Mn-based redox activity to efficiently scavenge highly toxic ROS via dual-enzyme-mimetic reactions, thereby attenuating TGF-β/Smad signaling and suppressing HSC activation. Concurrently, the catalytic generation of O_2_ alleviates liver hypoxia, synergistically enhancing the anti-fibrotic efficacy. In summary, this work proposes an integrated therapeutic strategy that couples homologous activated HSCs targeting with catalytic microenvironment remodeling to break the fibrogenic feedback loop and ameliorate liver fibrosis.

**Figure 1:**
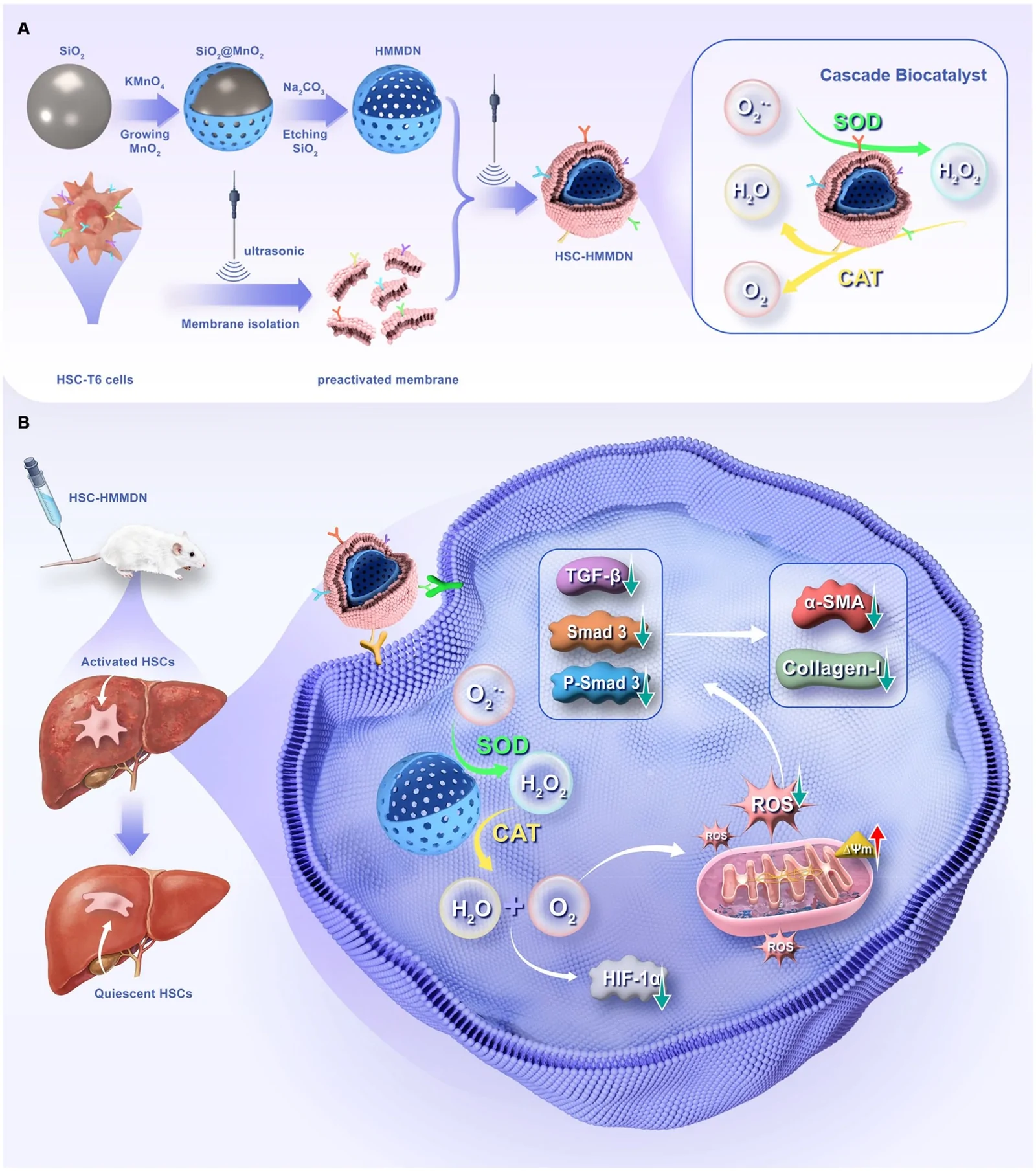
Schematic for the synthesis of HSC-HMMDN and their working mechanism in liver fibrosis treatment. (**A**) The preparation process for HMMDN NPs based on the hard-template method; coating HMMDN NPs with HSC-Mem through ultrasonication technique enhances their catalytic efficiency, biocompatibility and cellular internalization. (**B**) Following internalization, HSC-HMMDN exhibit intrinsic SOD- and CAT-mimetic activities, scavenging intracellular ROS and alleviating hypoxia. This microenvironmental remodeling suppresses activation of the TGF-β/Smad signaling pathway and inhibits collagen secretion, thereby alleviating the degree of liver fibrosis in rats.

## Materials and methods

### Materials and reagents

Rat HSC line HSC-T6 and rat hepatocyte cell line BRL were obtained from Procell Life Science & Technology Co., Ltd. (Shanghai, China). 1,2-Dipalmitoyl-sn-glycerol-3-phosphatidylcholine (DPPC) and 1,2-dipalmitoyl-sn-glycerol-3-phosphoethanolamine-polyethylene glycol-2000 (DPPE-mPEG2000) were obtained from Aladdin Biochemical Technology Co., Ltd. (Shanghai, China). Tetraethyl orthosilicate (TEOS), N-cetyltrimethylammonium bromide (CTAB), potassium permanganate (KMnO_4_), sodium carbonate (Na_2_CO_3_) and olive oil were purchased from Shanghai Macklin Biochemical Technology Co., Ltd. (Shanghai, China). 2,2-Diphenyl-1-picrylhydrazyl (DPPH) free radical scavenging capacity assay kit, 2,2′-azinobis-(3-ethylbenzthiazoline-6-sulfonic acid) (ABTS) free radical scavenging capacity assay kit, superoxide anion (O_2_•^-^) activity content assay kit, superoxide dismutase (SOD) activity assay kit, catalase (CAT) activity assay kit and the Cell Counting Kit-8 (CCK-8) were procured from Solarbio Technology Co., Ltd. (Beijing, China). 2′,7′-Dichlorodihydrofluorescein diacetate (DCFH-DA) and the mitochondrial membrane potential assay kit with JC-1 were purchased from Abbkine Scientific Co., Ltd. (Wuhan, China). IR-780, DAPI and DiI were obtained from GLPBIO Technology Co., Ltd. (Shanghai, China). Carbon tetrachloride (CCl_4_) was purchased from Rhawn Technology Co., Ltd. (Shanghai, China). The phosphatase and protease inhibitor cocktail, as well as the BCA protein assay kit, were supplied by Epizyme Biotechnology Co., Ltd. (Shanghai, China). Detailed antibody information has been listed in Supplementary Table S1.

### Extraction of HSC membranes

The HSC-T6 cells were first stimulated with 5 ng/mL TGF-β1 in Dulbecco’s Modified Eagle’s medium (DMEM) containing 1% fetal bovine serum (FBS) for 12 h to induce activation. Cells were harvested by centrifugation (600 *g*, 4°C, 3 min), washed twice with PBS and resuspended in a buffer containing 10 mM Tris, 10 mM MgCl_2_ and protease inhibitor. The suspension was subjected to ultrasonication in an ice bath to disrupt the cell membranes and release membrane components, followed by centrifugation at 2000 *g* for 10 min at 4°C to remove nuclei and debris. The supernatant was then centrifuged at 12 000 × *g* for 30 min at 4°C to collect the cell membranes. The resulting membrane pellet was washed twice with PBS to remove unbound soluble proteins and other impurities and stored at −80°C for later use.

### Synthesis of Lip-HMMDN and HSC-HMMDN

Solid silica (SiO_2_) nanoparticles (NPs) were synthesized via the Stöber method [44]. Using SiO_2_ as a hard template, we reacted them with KMnO_4_ to form a uniform MnO_2_ coating, yielding SiO_2_@MnO_2_. The SiO_2_ core was selectively etched by treatment with Na_2_CO_3_ solution, resulting in HMMDN NPs. The prepared HMMDN was mixed with HSC membrane (HSC-Mem) at a mass ratio of 1:10. The mixture was treated with an intelligent ultrasonic processor (Shunma YQ-100, China, 100 W, 5 min) in an ice bath to facilitate membrane fusion onto the NPs surface. The resulting HSC-HMMDN were purified by three rounds of centrifugation (12 000 *g*, 10 min, 4°C) to remove excess free membranes. The liposome-coated NPs were obtained using the thin-film hydration method combined with ultrasonic emulsification. The mixing ratio of DPPC: DPPE-mPEG2000: DC-Chol was 5: 1: 0.5. Then, HMMDN and the liposomes were mixed at a mass ratio of 1:10. After three rounds of centrifugation for purification, Lip-HMMDN were obtained.

### Characterizations

The morphology of SiO_2_@MnO_2_ and HMMDN NPs was investigated by transmission electron microscopy (TEM, Hitachi HT7700, Japan). The elemental distribution of the HSC-HMMDN was assessed using energy dispersive X-ray spectroscopy (EDS, JEOL-JEM-F200, Japan). The average hydrodynamic diameter and zeta potential of HMMDN, HSC-Mem and HSC-HMMDN were determined via Malvern Zetasizer Nano ZS (Malvern Instruments, Malvern, UK). Successful coating of HSC-Mems onto HMMDN NPs was verified by Coomassie Brilliant Blue staining. Nitrogen adsorption/desorption isotherms of HMMDN were measured with a Micromeritics TriStar II Plus surface area and pore size analyzer, and the specific surface area values and pore size distribution curves were obtained by Brunauer–Emmett–Teller (BET) and Barrett–Joyner–Halenda (BJH) methods, respectively (Micromeritics Instrument Corporation, Norcross, GA, USA). X-ray diffraction (XRD, Bruker AXS GmbH, Karlsruhe, Germany) was used to measure the crystal structures of HMMDN. The surface elemental composition and chemical valence states were further analyzed by X-ray Photoelectron Spectroscopy (XPS, Thermo Fisher Scientific, Waltham, MA, USA).

### Dual-enzyme-like antioxidative activity of HSC-HMMDN

The CAT-like activity of HSC-HMMDN was assessed by measuring H_2_O_2_ decomposition using the titanium sulfate colorimetric method. Briefly, a mixture of 20 μL of 0.1 M H_2_O_2_, 1.97 mL of PBS and 10 μL of HSC-HMMDN at different concentrations (0, 20, 40, 60, 80 and 100 μg/mL) was prepared and reacted for 60 min. Every 10 min, 100 μL of the mixture was combined with 100 μL of Ti (SO_4_)_2_ solution (13.9 mM), and the absorbance at 405 nm was measured using a microplate reader (SpectraMax iD3, Molecular Devices, USA). Next, a portable dissolved oxygen meter (JPBJ-608, China) was used to dynamically measure the level of dissolved oxygen produced by the decomposition of H_2_O_2_. Three groups were set up: (1) H_2_O_2_ (1 mM), (2) HSC-HMMDN (100 μg/mL) and (3) HSC-HMMDN (100 μg/mL) + H_2_O_2_ (1 mM). The dissolved oxygen content was recorded every minute for 30 min. SOD-like activity was evaluated using the WST-1 method, which is based on the competitive inhibition of formazan formation. Different concentrations of HSC-HMMDN (0, 20, 40, 60, 80 and 100 μg/mL) were incubated with the WST-1 working solution following the manufacturer’s protocol. Absorbance at 450 nm was recorded. The O_2_•^-^ scavenging capacity assay kit was used to measure the scavenging of O_2_•^-^.

### Radical scavenging experiment

To evaluate the radical scavenging activity, the HSC-HMMDN nanozyme was incubated with ABTS and DPPH working solutions according to the manufacturers’ instructions. The absorbance of the reaction mixture was then measured at 734 nm for the ABTS assay and at 517 nm for the DPPH assay using a microplate reader. The scavenging rates were calculated using the following equation:


$$\text{Scavenging}\;\text{rate}\;(\%)\;=\;[(A_{0}-A_{i})/A_{0}]\;\times \;100\%.$$

Where *A*_0_ is the absorbance of the blank control (radical solution without nanozymes) and *A_i_* is the absorbance of the test sample after reaction.

### Cell culture and model establishment

HSC-T6 cells were cultured in DMEM supplemented with 10% FBS and 1% penicillin‑streptomycin at 37°C in a humidified atmosphere containing 5% CO₂. To induce HSC activation, the medium was replaced with fresh DMEM containing 5 ng/mL TGF‑β1 and cells were incubated for 12 h. For the inhibition experiments, after TGF-β1 pre-stimulation, the medium was replaced with fresh DMEM containing HSC‑HMMDN or Lip‑HMMDN. Cells incubated with DMEM without TGF-β1 or NPs served as negative control.

### *In vitro* targeting and subcellular localization of HSC-HMMDN

DiI-labeled HSC-HMMDN and Lip-HMMDN (100 μg/mL) were used to establish three experimental groups: (a) HSC-HMMDN with HSC-T6 cells; (b) Lip-HMMDN with HSC-T6 cells; (c) HSC-HMMDN with BRL cells. Cell nuclei were stained with DAPI. Cellular uptake was assessed by confocal laser scanning microscopy (CLSM; Olympus, Tokyo, Japan) after 12 or 24 h of co-incubation, and the signal intensity was quantified using Image J software [National Institutes of Health (NIH), Bethesda, MD, USA]. To further validate the homologous targeting mechanism, DiI-labeled HSC-HMMDN underwent 1-h incubation with adhesion-specific blocking antibodies (including anti-E-cadherin, anti-N-cadherin, anti-integrin av, anti-integrin β1 and anti-integrin β3 antibodies). Then, the pretreated HSC-HMMDN were co-incubated with HSC-T6 cells for 12 h. Flow cytometry (Olympus Corporation, Japan) was performed to observe fluorescence intensity before and after antibody pretreatment. For subcellular localization, HSC-T6 cells were incubated with different concentrations (50 and 100 μg/mL) of DiI-labeled HSC-HMMDN for 12 h. Mitochondria were then labeled with an antibody specific to the outer mitochondrial membrane (Tom20). Following immunofluorescence staining, the co-localization of NPs and mitochondria was observed under a CLSM.

### *In vitro* antioxidant effects of HSC-HMMDN

HSC-T6 cells were plated in confocal dishes (5 × 10^4^) for 12 h. Cells were then incubated with HSC-HMMDN or Lip-HMMDN for 24 h after TGF-β1 (5 ng/mL) pre-stimulation. For intracellular ROS detection, cells were stained with DCFH-DA probe for 30 min. After washing with PBS three times, cells were analyzed by CLSM and flow cytometry. For JC-1 staining, cells were incubated with JC-1 dye for 20 min, washed with PBS, and then imaged by CLSM. The monomer/aggregate ratio reflects membrane polarization status. For intracellular oxygen level measurement, HSC-T6 cells were treated with CoCl_2_ (100 μM, 12 h) to induce hypoxia. The pre-treated cells were then incubated with HSC-HMMDN or Lip-HMMDN for another 24 h. Next, cells were stained with [Ru(dpp)_3_]Cl_2_ (10 μM) at 37°C for 6 h and analyzed by flow cytometry.

### Cell migration ability detection

HSC-T6 cells were plated in 6-well plates at a density of around 5 × 10^5^ cells per well. After the cells adhered and formed a thin monolayer, they were scratched with the tip of a sterile pipette. Subsequently, the cells were washed with PBS to eliminate any remaining cell debris. After different treatments (control, TGF-β1, Lip-HMMDN and HSC-HMMDN), cell scratch wounds underwent imaging at 0, 12 and 24 h, followed by calculation of the wound healing rate.

### Immunofluorescence staining

Cell samples were fixed with 4% paraformaldehyde for 10 min at room temperature. After rinsing with PBS, the samples were blocked with 3% bovine serum albumin (BSA) for 20 min to minimize nonspecific binding. Subsequently, the specimens were incubated overnight at 4°C with primary antibodies diluted in blocking buffer. Following three washes with PBS, the corresponding fluorophore-conjugated secondary antibodies were applied for 1 h at 25°C under dark conditions. Finally, DAPI nuclear staining (10 min) was applied.

### RNA sequencing

HSC‑T6 cells were seeded into 6-well plates at a density of 1 × 10^5^ cells per well and incubated for 24 h. Cells were then divided into two groups: TGF‑β1 and TGF‑β1 + HSC‑HMMDN. After treatment, cells were washed twice with cold PBS and harvested. Total RNA was extracted using TRIzol reagent. The obtained RNA was subjected to transcriptome sequencing and bioinformatics analysis.

### Real-time quantitative PCR

Total cellular RNA was extracted using RNAex Pro reagent (Accurate Biology, China) following the manufacturer’s protocol, and its concentration was then determined with a Nanodrop 2000C (Thermo Scientific, USA). Following the removal of genomic DNA (gDNA) from the RNA samples, reverse transcription was carried out using the Evo M-MLV RT hybrid kit (Accurate Biotechnology, China) to synthesize cDNA. Subsequently, the resulting cDNA was subjected to amplification with the SYBR® Green Premix Pro-Taq HS qPCR Kit (Accurate Biotechnology, China) and sequence-specific primers on the CFX96 real-time PCR detection platform (Bio-Rad, USA). Supplementary Table S2 presents the primer sequences, and the relative expression levels were determined via the 2^-ΔΔCt^ method.

### Animal study

Ethical approval for all animal studies was granted by the Ethics Committee of The Southwest Jiaotong University [SWJTU-2203-NSFC (029)]. SD rats (male, 4–6 weeks old, 200 ± 20 g) were obtained from Chengdu Huafukang Experimental Animal Co., Ltd., China. All animal care and experimental procedures were conducted in accordance with the guidelines of the Ethics Committee of The Southwest Jiaotong University. In our model, liver fibrosis was induced by 4-week subcutaneous administration of 40% CCl_4_ (in olive oil, 5 mL/kg) thrice weekly to rats. Rats were randomly divided into four groups: the control, model, Lip-HMMDN and HSC-HMMDN groups. The control group consisted of healthy rats, serving as the negative control. The model group comprised rats with CCl_4_-induced liver fibrosis that received no further treatment, serving as the positive control. Rats in the drug-treated group received tail vein injections of NPs (2 mg/kg, injection volume: 1 mL) every 3 days for 4 weeks, whereas the control group was administered an equivalent volume of 0.9% normal saline. The model group and treatment groups were maintained on CCl_4_/olive oil injections throughout the treatment period. Blood and major organs were collected 4 weeks after the first NPs treatment. Serological tests were used to evaluate liver function by measuring serum levels of albumin (ALB), alkaline phosphatase (ALP), aspartate transaminase (AST) and alanine transaminase (ALT). Tissue samples were preserved for histopathological analysis.

### *In vivo* targeting

To enable *in vivo* imaging, we labeled HSC-HMMDN and Lip-HMMDN with a near-infrared-absorbing cyanine dye, IR780. Through the tail vein, IR780-labeled HSC-HMMDN and Lip-HMMDN were administered to fibrotic rats (IR780 dose: 2 mg/kg). Noninvasive molecular imaging of rats was performed at various intervals using a Xenogen IVIS-200 *in vivo* imaging system (Xenogen Corp., Alameda, CA, USA) with suitable excitation and emission settings applied to capture the fluorescence signals. Subsequently, major organs were excised for ex vivo imaging analysis.

### Histological analysis

Following fixation in 10% formalin, tissues underwent processing, dehydration and paraffin embedding. The obtained paraffin-embedded blocks were cut into thin slices of 4 µm. Subsequent to deparaffinization and hydration, staining was performed on the sections. Pathological evaluation of tissue organization employed H&E staining, whereas collagen content was quantified using Masson staining and Sirius red staining. Randomly selected fields within stained sections were photographed microscopically.

### Multiplex immunohistochemistry

Formalin-fixed, paraffin-embedded liver sections (4 μm) were deparaffinized in xylene and rehydrated via a graded ethanol series. Heat-induced epitope retrieval was performed in EDTA buffer (pH 9.0) at 95°C for 20 min, followed by cooling. Endogenous peroxidase was blocked with 3% H_2_O_2_ for 10 min, and nonspecific binding was blocked with 3% BSA for 30 min. Then, sections were sequentially treated with primary antibodies against α-SMA, collagen I and HIF-1α overnight at 4°C. After each primary antibody, HRP-conjugated secondary antibodies were applied for 30 min at room temperature, followed by tyramide signal amplification using Opal fluorophores (520, 570, 690) for 10 min. Between cycles, antibodies were stripped by microwave treatment in EDTA buffer (800 W, 3 min). Finally, nuclei were counterstained with DAPI for 5 min.

### Western blot analysis

Total protein was extracted from harvested cells and liver tissues using pre-chilled RIPA lysis buffer supplemented with protease inhibitors. After centrifugation at 13 000 × *g* for 15 min at 4°C, the protein concentration in the supernatants was determined using a BCA protein assay kit. Equal amounts of protein were separated by SDS-PAGE and transferred onto polyvinylidene fluoride (PVDF) membranes. The membranes were blocked with 5% non-fat milk for 2 h at room temperature, followed by overnight incubation at 4°C with primary antibodies. Subsequently, the membranes were incubated with corresponding secondary antibodies for 1 h at 25°C. Protein bands were visualized using an enhanced chemiluminescence kit, followed by densitometric analysis with ImageJ software.

### Biocompatibility evaluation

For the CCK-8 assay, BRL cells (1 × 10⁴ cells/well) were seeded in 96-well plates with 100 µL 10% FBS medium. Then, the original medium was replaced with fresh medium containing varying concentrations of HSC-HMMDN or Lip-HMMDN and the cells were incubated for 24 h. Finally, 10 μL of CCK-8 solution (10%) was added to each well, followed by 1 h incubation. The optical density (OD) at 450 nm was measured using a microplate reader.

For hemolytic test, whole blood was collected from the orbital venous plexus of healthy SD rats and centrifuged (5000 rpm, 5 min). The pelleted erythrocytes were resuspended in PBS, then incubated with HSC-HMMDN. Deionized water and saline solution served as positive and negative controls, respectively. After 1 h of incubation at 37°C, the mixtures were re-centrifuged for subsequent imaging analysis. Supernatant absorbance at 570 nm was measured via microplate reader. Hemolysis percentage was determined with the formula:


Hemolysis (%) = (ODsample−ODnegative)/(ODpositive−ODnegative) × 100%.

After administering different treatments (saline, Lip-HMMDN and HSC-HMMDN) to healthy rats for 4 weeks, serum levels of ALB, ALP, ALT and AST were detected to evaluate their impact on liver function. Additionally, major organs were collected from the fibrotic rats in each group (treated with saline, Lip-HMMDN or HSC-HMMDN). All tissue sections were subjected to H&E staining for histopathological examination. Apoptotic cells were subsequently identified via terminal deoxynucleotidyl transferase dUTP nick end labeling (TUNEL) staining, as evidenced by green fluorescence.

### Statistical analyses

Data are presented as mean ± SD. Comparisons between two groups were performed using Student’s *t*-test. Multiple group comparisons were analyzed by one-way analysis of variance (ANOVA). Statistical significance was denoted as follows: * *P *< 0.05, ** *P *< 0.01, *** *P *< 0.001, **** *P *< 0.0001. All statistical analyses were conducted with GraphPad Prism software (version 10.1.2).

## Ethics approval and consent to participate

Ethical approval for all animal studies was granted by the Ethics Committee of the Southwest Jiaotong University [SWJTU-2203- NSFC (029)]. All animal care and experimental procedures were conducted in accordance with the guidelines of the Ethics Committee of the Southwest Jiaotong University.

## Results and discussion

### Characterization of HSC-HMMDN

TEM measurement revealed that both SiO_2_@MnO_2_ and HMMDN NPs exhibited a spherical morphology with diameters ranging from 100 to 120 nm (Figure 2A and B). The hollow structure of HMMDN was obtained by dissolving SiO_2_ in the Na_2_CO_3_ solution. The mesoporous shells had a thickness of ∼8 nm. Dynamic light scattering (DLS) analysis showed that the hydrodynamic diameter of HSC-HMMDN (121.1 ± 4.7 nm) was moderately larger than that of bare HMMDN (107.6 ± 6.4 nm), while the surface zeta potential shifted from −23.6 ± 1.9 mV (HMMDN) to −31.3 ± 0.2 mV (HSC-HMMDN; Figure 2C). These changes were attributable to the presence of the negatively charged HSC-Mem coating. The hydrodynamic diameter distribution of HSC-HMMDN was shown in Supplementary Figure S1, indicating good dispersion. Satisfyingly, the HSC-HMMDN exhibited considerable stability, with negligible changes in diameter (± 10 nm) and zeta potential (± 5 mV) over the 7-day observation period (Supplementary Figure S2).

**Figure 2:**
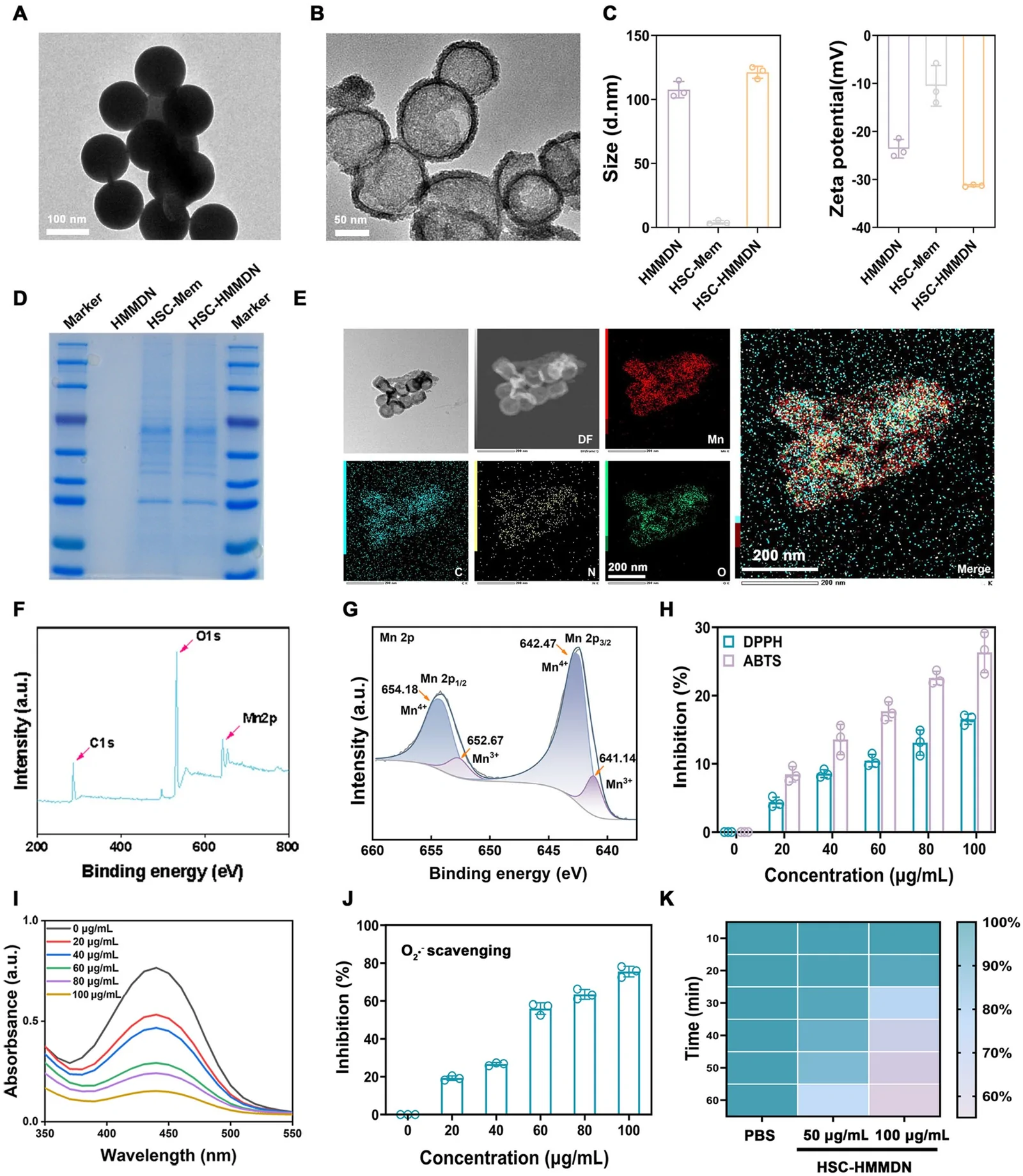
Characterization of NPs. Representative TEM images of SiO_2_@MnO_2_ NPs (**A**) and HMMDN NPs (**B**). (**C**) The hydrodynamic sizes and zeta potentials of HMMDN NPs, HSC-Mem and HSC-HMMDN. (**D**) Total membrane proteins for HMMDN NPs, HSC-Mem and HSC-HMMDN. (E) TEM EDS mapping images of HSC-HMMDN. Scale bar: 200 nm. (**F**) XPS survey spectrum of HMMDN NPs. (**G**) High-resolution Mn 2p XPS spectrum of HMMDN NPs. (**H**) ABTS and DPPH scavenging capacity of HSC-HMMDN. (**I**) Wavelength scan of SOD-like activity at different concentrations of HSC-HMMDN. (**J**) O_2_•^-^ scavenging ability of HSC-HMMDN. (**K**) CAT-like activity at various times and concentrations of HSC-HMMDN. Data represent mean ± SD (*n* = 3).

To investigate the integrity of membrane protein retention, we performed a Coomassie Brilliant Blue staining. As shown in Figure 2D, bare HMMDN exhibited no detectable protein bands. Conversely, HSC-HMMDN displayed a protein profile closely matching with HSC-Mem lane, with both enriched solely in specific membrane protein bands, indicating the successful grafting of membrane proteins onto the NPs surface. This intact protein coating is essential for the homologous targeting capability of HSC-HMMDN, as it preserves the native membrane architecture and receptor repertoire required for specific recognition of activated HSCs. EDS mapping analysis in Figure 2E revealed the coexistence of Mn, C, O and N elements, confirming the successful fabrication of the HSC-HMMDN.

HMMDN was analyzed by the nitrogen adsorption-desorption isotherm to assess its porosity (Supplementary Figure S3). The BET specific surface area was 26.2803 m^2^/g, and total pore volume reached 0.056034 cm^3^/g. The BJH pore size distribution exhibited a concentrated pore volume within the mesopore range (2–50 nm), confirming the mesoporous presence of the materials. As illustrated in Supplementary Figure S4, the XRD pattern of HMMDN was compared with that of standard MnO_2_ (JCPDS No. 42-1169), and the two diffraction peaks at 36.9° and 65.3° were found to be highly overlapped, confirming the form of MnO_2_ as γ-MnO_2_. XPS analysis confirmed the surface chemical composition of the synthesized HMMDN, as shown in Figure 2F. The high-resolution Mn 2p XPS spectrum of HMMDN was presented in Figure 2G. The spectrum was decomposed into four peaks at 641.14 eV (Mn^3+^), 642.47 eV (Mn^4+^), 652.67 eV (Mn^3+^) and 654.18 eV (Mn^4+^). The quantitative analysis reveals that the surface Mn^4+^ and Mn^3+^ contents are 86.34% and 13.66%, respectively, confirming the coexistence of multiple valence states.

### *In vitro* antioxidant and enzyme-mimicking activities of HSC-HMMDN

Excessive ROS play a critical role in triggering oxidative stress [45]. In HSC-HMMDN nanozymes, the hollow mesoporous structure of MnO_2_ provides more active sites, facilitating efficient contact with free radicals and rapid electron transfer, thereby enhancing ROS scavenging efficiency. To verify this, we first evaluated the radical-scavenging capacity of HSC-HMMDN using DPPH and ABTS. As shown in Figure 2H, HSC-HMMDN scavenged both DPPH and ABTS radicals in a concentration-dependent manner, achieving scavenging rates of 16.5% ± 0.7% and 26.3% ± 3.0%, respectively, at 100 μg/mL. These results demonstrate that HSC-HMMDN possesses electron-donating ability and intrinsic antioxidant activity, likely attributable to the surface Mn^4+^/Mn^3+^ redox dynamics characteristic of MnO_2_-based nanozymes.

Building on these findings, we next assessed its specific enzyme-mimicking functions using WST-1 and titanium sulfate assays. O_2_•^-^ serves as the initiator of radical chain reactions and is the principal substrate for SOD enzymes [46]. As depicted in Figure 2I, HSC-HMMDN induced a concentration-dependent decrease in absorbance, demonstrating robust SOD-mimicking activity. The activity increased steeply over the concentration range of 20-60 μg/mL and reached an O_2_•^−^ scavenging rate of 75.5 ± 2.8% at 100 μg/mL (Figure 2J), indicating high catalytic efficiency. Furthermore, HSC-HMMDN catalyzed H_2_O_2_ decomposition in a time- and concentration-dependent manner, confirming its CAT-mimicking activity (Figure 2K). Then, we studied the capacity of HSC-HMMDN to catalyze the generation of O_2_ from H_2_O_2_. As presented in Supplementary Figure S5, the catalytic reaction was markedly enhanced compared to the controls, with dissolved O_2_ increasing rapidly from 7.88 mg/L to 13.42 mg/L within 5 min. These findings confirm the strong oxygen‑generating capacity of HSC-HMMDN, likely due to its CAT‑like activity. Collectively, these results establish HSC-HMMDN as a broad-spectrum antioxidant nanozyme capable of efficiently eliminating O_2_•^−^, H_2_O_2_, ABTS and DPPH, thereby providing a molecular rationale for its application in oxidative stress-associated conditions.

### *In vitro* and *in vivo* targeting efficacy of HSC-HMMDN

To evaluate the targeting efficacy of HSC-HMMDN, we synthesized Lip-HMMDN as a control. Since TGF-β1 serves as a predominant pro-fibrotic cytokine that drives HSCs transdifferentiation into α-SMA-positive myofibroblasts, we employed TGF-β1 to activate quiescent HSC-T6 cells into a fibrogenic phenotype. As presented in Figure 3A, the intensity of DiI-labeled red fluorescence increased progressively with prolonged incubation. After 24 h of incubation, the uptake of Lip-HMMDN in activated HSC-T6 cells was similar to that of HSC-HMMDN in BRL hepatocytes. In contrast, the uptake of HSC-HMMDN in activated HSC-T6 cells was approximately 11.7-fold higher than that of Lip-HMMDN (Supplementary Figure S6). This enhanced cellular uptake is ascribed to the presence of HSC-Mem. Furthermore, HSC-HMMDN uptake in activated HSC-T6 cells was approximately 7.7-fold higher than in BRL hepatocytes, confirming the specificity of HSC-Mem-mediated homologous targeting.

**Figure 3:**
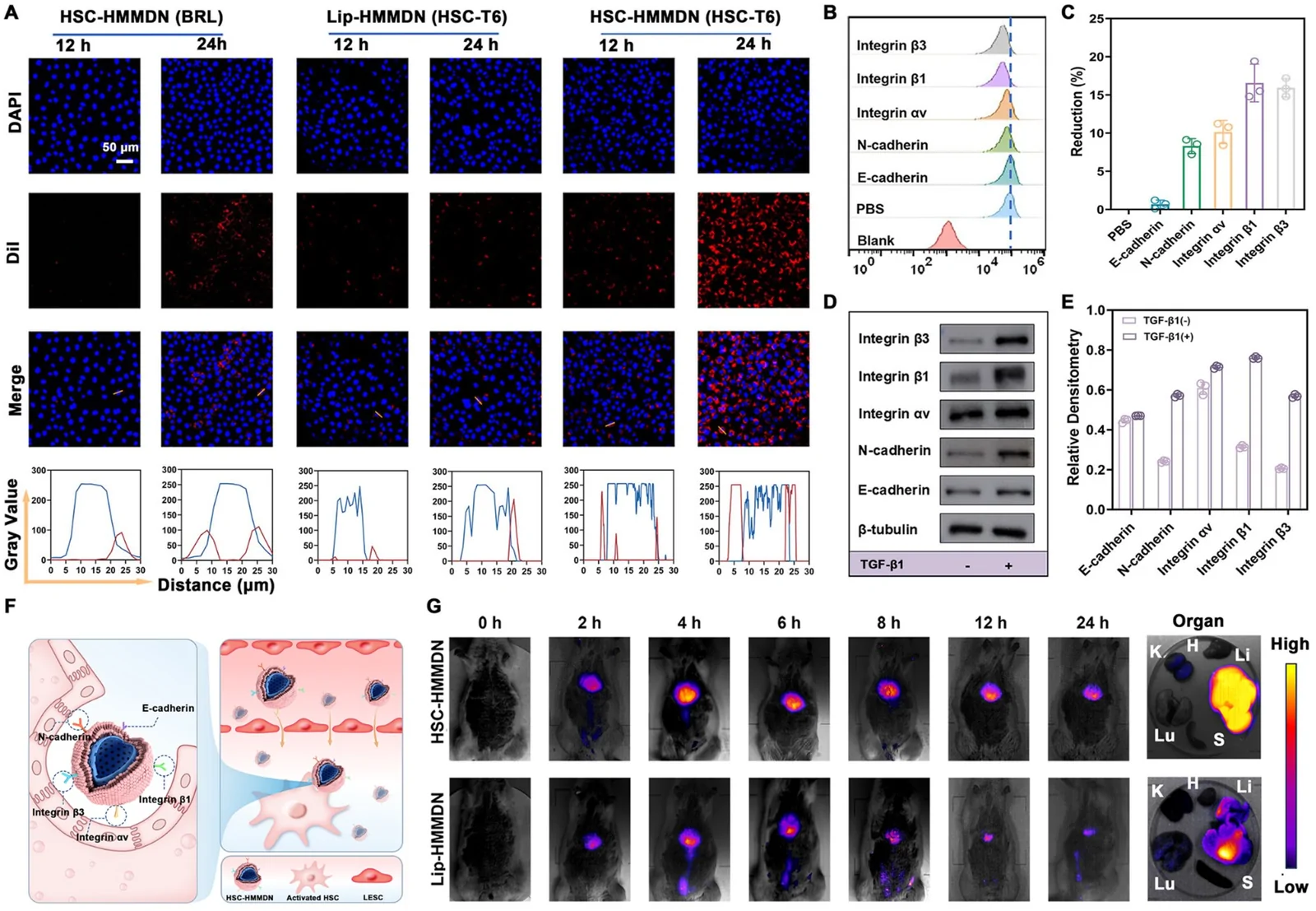
Targeting capability and biodistribution of HSC-HMMDN. (**A**) CLSM images and the corresponding linear-scan profiles of cells after co-incubation for 12 and 24 h with DiI-labeled HSC-HMMDN and Lip-HMMDN. Scale bar: 50 μm. (**B**) Flow cytometry analysis of the homologous targeting mechanism of DiI-labeled HSC-HMMDN in HSCs through pre-incubation for 1 h using specific antibodies. (**C**) Quantitative analysis of the corresponding mean fluorescence intensity in (**B**). (**D**) Western blot assessment of E-cadherin, N-cadherin, integrin αv, integrin β1 and integrin β3 in quiescent HSCs and activated HSCs. (**E**) Quantitative analysis of the protein expression levels in (**D**). (**F**) Diagram of the targeting process for HSC-HMMDN. (**G**) *In vivo* fluorescence images of CCl_4_-induced liver fibrotic rats were obtained at various time points, and *ex vivo* images of the dissected major organs were captured at 24 h after injection of DiI-labeled HSC-HMMDN or Lip-HMMDN. Data represent mean ± SD (*n* = 3). LSEC: liver sinusoidal endothelial cell.

The high targeting specificity of HSC-HMMDN NPs is likely attributable to adhesion proteins expressed on the HSC-Mem. Growing evidence suggests that integrin and cadherin, which are key mediators of cell adhesion, are highly upregulated during HSC activation, especially integrin αv, β1 and β3, as well as N-cadherin [42, 47]. To elucidate the mechanism underlying this homologous targeting, we pre-incubated HSC-HMMDN with blocking antibodies against E-cadherin, N-cadherin, integrin αv, integrin β1 and integrin β3. As displayed in Figure 3B and C, fluorescence intensity was significantly reduced following blockade with anti-integrin αv (10.1% ± 1.5%), anti-integrin β1 (16.6% ± 2.5%), anti-integrin β3 (15.9% ± 1.2%) and anti-N-cadherin (8.3% ± 1.0%). Consistent with these findings, western blot analysis confirmed that integrins αv, β1, β3 and N-cadherin were abundantly expressed on activated HSC-Mem, whereas E-cadherin expression remained low (Figure 3D and E). Together, these results strongly support the involvement of specific integrins and N-cadherin in the membrane-mediated homologous targeting of HSC-HMMDN to activated HSCs. This multi-ligand targeting strategy confers superior specificity and efficiency over conventional mono-ligand nanosystems.

To assess the *in vivo* biodistribution and clearance of HSC-HMMDN, CCl_4_-induced fibrotic rats were intravenously injected with IR780-labeled HSC-HMMDN and Lip-HMMDN for fluorescence tracking. At 4 h after administration, IR780-labeled Lip-HMMDN showed significant fluorescence in the bladder and liver. The bladder signal suggests efficient renal clearance, while the liver signal indicates substantial uptake due to the natural trapping of NPs by the reticuloendothelial system (Figure 3G and Supplementary Figure S7). Notably, the fluorescence had weakened considerably at 24 h. By contrast, the IR780-labeled HSC-HMMDN were primarily distributed to the fibrotic liver, with negligible distribution in other organs at all time points following administration. A substantial signal was still detectable even after 24 h, indicating a prolonged circulation time *in vivo*. Furthermore, the major organs were collected from the rats and subjected to *ex vivo* fluorescence imaging. Compared with IR780-labeled Lip-HMMDN, the accumulation of HSC-HMMDN in the fibrotic liver was significantly higher. Quantitative analysis further confirmed that the integrated fluorescence signal in the liver was 2.85-fold higher in rats with IR780-labeled HSC-HMMDN treatment than with IR780-labeled Lip-HMMDN (Supplementary Figure S8). In summary, these findings demonstrate that HSC-HMMDN achieves rapid, sustained and highly selective delivery to the fibrotic liver and that HSC-Mem decoration significantly enhances the targeting specificity and efficiency of HMMDN.

### *In vitro* antioxidant performance of HSC-HMMDN

During liver injury, excessive ROS in activated HSCs act as key intracellular signals that promote HSC activation and stimulate the overproduction of ECM components. Based on the excellent aforementioned dual SOD/CAT-mimicking activities of the HSC-HMMDN nanozyme, we next analyzed the effect of HSC-HMMDN on intracellular ROS elimination. There was a notable increase in green fluorescence in activated HSCs stimulated by TGF-β1, suggesting an augmentation of ROS production (Figure 4A). Treatment with Lip-HMMDN reduced the fluorescence intensity by approximately 2.3-fold, whereas HSC-HMMDN achieved a more pronounced 8.5-fold reduction. Importantly, HSC-HMMDN treatment led to over 70% lower ROS levels compared to Lip-HMMDN, implying that the HSC-Mem coating efficiently enhances HMMDN-related ROS elimination through homologous targeting-mediated cellular uptake (Supplementary Figure S9). These findings were further corroborated by flow cytometry analysis (Figure 4B). Besides ROS scavenging capacity, we also investigated its effect on intracellular oxygen levels. As shown in Figure 4C, the [Ru(dpp)_3_]Cl_2_ fluorescence intensity was significantly decreased in the HSC‑HMMDN‑treated group compared to CoCl_2_ group, indicating that HSC‑HMMDN effectively elevates intracellular oxygen levels and alleviates hypoxia. Together, these findings demonstrate the superior capacity of HSC‑HMMDN to alleviate oxidative stress.

**Figure 4:**
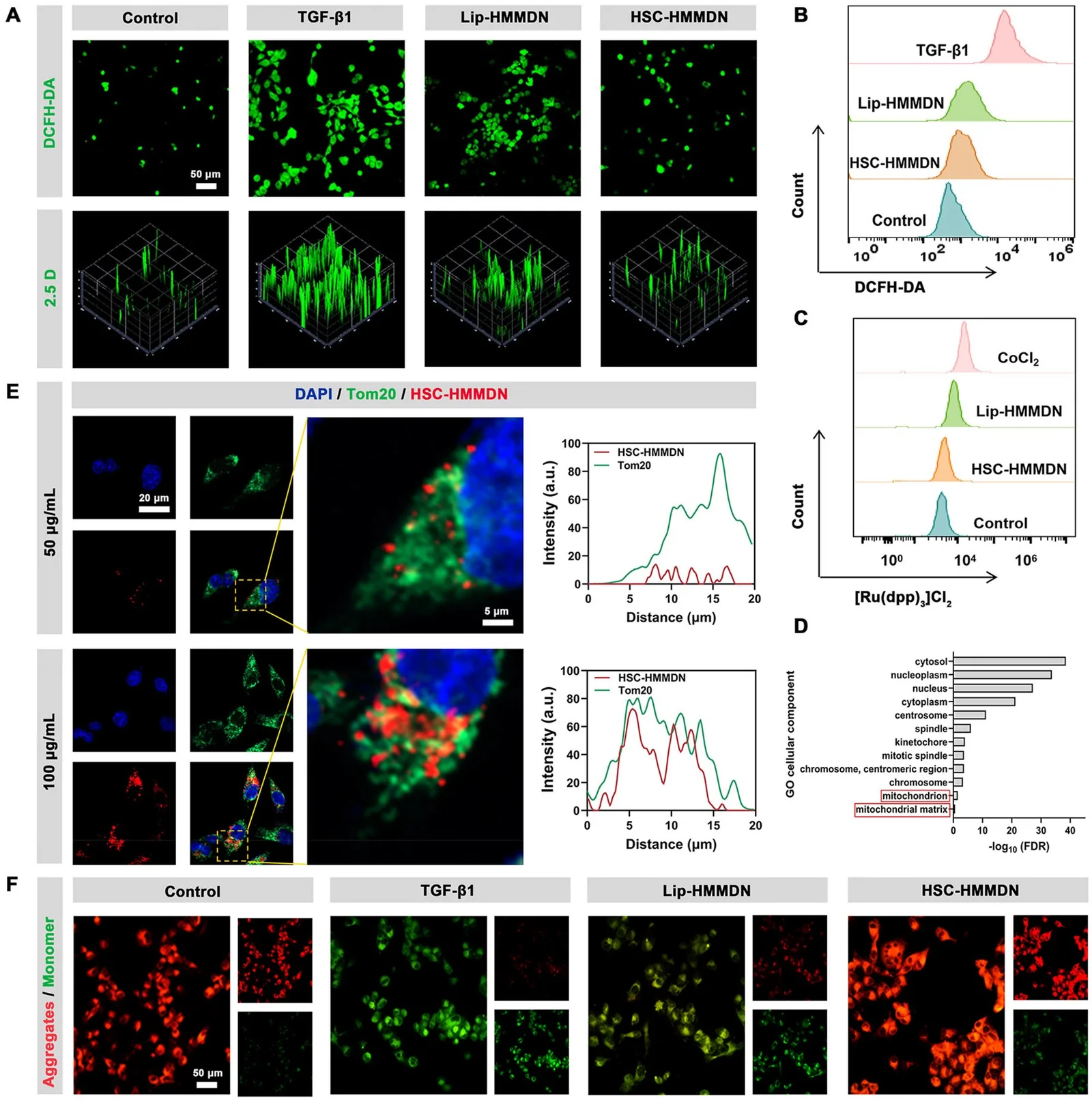
*In vitro* antioxidant ability of HSC-HMMDN. (**A**) Detection of intracellular ROS in the HSC-T6 cells incubation with TGF-β1 and treated with Lip-HMMDN and HSC-HMMDN, respectively, using DCFH-DA. The scale bar: 50 μm. (**B**) Flow cytometry analysis of DCFH-DA fluorescence intensity in HSCs after various treatments. (**C**) Flow cytometry analysis of [Ru(dpp)_3_]Cl_2_ fluorescence intensity in HSC-T6 cells after different treatments. (**D**) GO enrichment analysis. (**E**) Subcellular localization of HSC-HMMDN at different concentrations in mitochondria. Scale bars: 20 μm and 5 μm. (**F**) Mitochondrial membrane potential of HSC-T6 cells incubation with TGF-β1 and treated with Lip-HMMDN and HSC-HMMDN, respectively. The scale bar: 50 μm.

Since intracellular ROS are primarily generated from the mitochondrial electron transport chain [48], and given that HSC‑HMMDN effectively scavenges ROS and alleviates hypoxia, we next investigated the influence of HSC‑HMMDN on mitochondria. GO cellular component (GO‑CC) enrichment analysis revealed that the differentially expressed genes were significantly enriched in the mitochondrion and mitochondrial matrix compartments, suggesting that HSC‑HMMDN treatment may affect mitochondrial‑related biological processes (Figure 4D). To determine whether this effect is associated with its specific subcellular localization, we assessed the spatial distribution of HSC‑HMMDN relative to mitochondria in HSC‑T6 cells. The resulting images showed clear co‑localization between the two signals (Figure 4E). Subsequently, we assessed whether HSC‑HMMDN could preserve mitochondrial function under oxidative stress. Mitochondrial membrane potential (MMP) was assessed using JC‑1 dye, which forms red fluorescent J‑aggregates in healthy mitochondria but reverts to green monomers upon depolarization. As illustrated in Figure 4F and Supplementary Figure S10, TGF‑β1 treatment induced a large shift from red aggregates to green monomers, indicating membrane disruption. This depolarization was substantially reversed by HSC‑HMMDN, which restored MMP to near‑normal levels, whereas Lip‑HMMDN showed only partial recovery. In summary, these results demonstrate that HSC‑HMMDN effectively restores mitochondrial membrane potential under oxidative stress conditions.

### The TGF-β/Smad is a key pathway for HSC-HMMDN to inhibit the activation and migration of HSC-T6 cells

The activation of HSCs, a pivotal event in liver fibrosis progression, is primarily regulated by the TGF-β/Smad signaling pathway [10]. Given that ROS overproduction can activate the TGF-β/Smad pathway, exacerbating ROS toxicity in activated HSCs and driving their activation, we next investigated whether the ROS-scavenging capability of HSC-HMMDN could interrupt this key fibrogenic signaling. Initially, we performed RNA sequencing analysis on HSC‑T6 cells treated with TGF‑β1 or TGF‑β1 + HSC‑HMMDN to elucidate its antifibrotic mechanism. KEGG enrichment analysis (Figure 5A) highlighted 10 key pathways, including the TGF‑β signaling pathway. To validate the transcriptomic findings, we examined mRNA and protein expression of TGF‑β/Smad pathway components using qRT‑PCR, Western blotting and immunofluorescence staining. As expected, TGF‑β1 stimulation significantly upregulated the mRNA levels of TGF‑β, Smad3, Serpine1, ACTA2 and Col1a1 (Figure 5B–F). Both Lip‑HMMDN and HSC‑HMMDN treatments reversed these upregulations, with HSC‑HMMDN showing markedly greater efficacy. Consistently, western blotting confirmed that HSC‑HMMDN more effectively reduced the protein levels of TGF‑β, Smad3, p‑Smad3, α‑SMA and collagen I compared with Lip‑HMMDN (Figure 5G and Supplementary Figure S11). Immunofluorescence staining further confirmed the downregulation of α‑SMA by HSC‑HMMDN (Figure 5H and Supplementary Figure S12). Collectively, these results demonstrate that HSC‑HMMDN effectively suppresses the TGF‑β/Smad signaling pathway and inhibits HSC activation. Moreover, the enhanced efficacy of HSC‑HMMDN over Lip‑HMMDN highlights the critical role of HSC-Mem‑mediated targeting in amplifying this antifibrotic effect.

**Figure 5:**
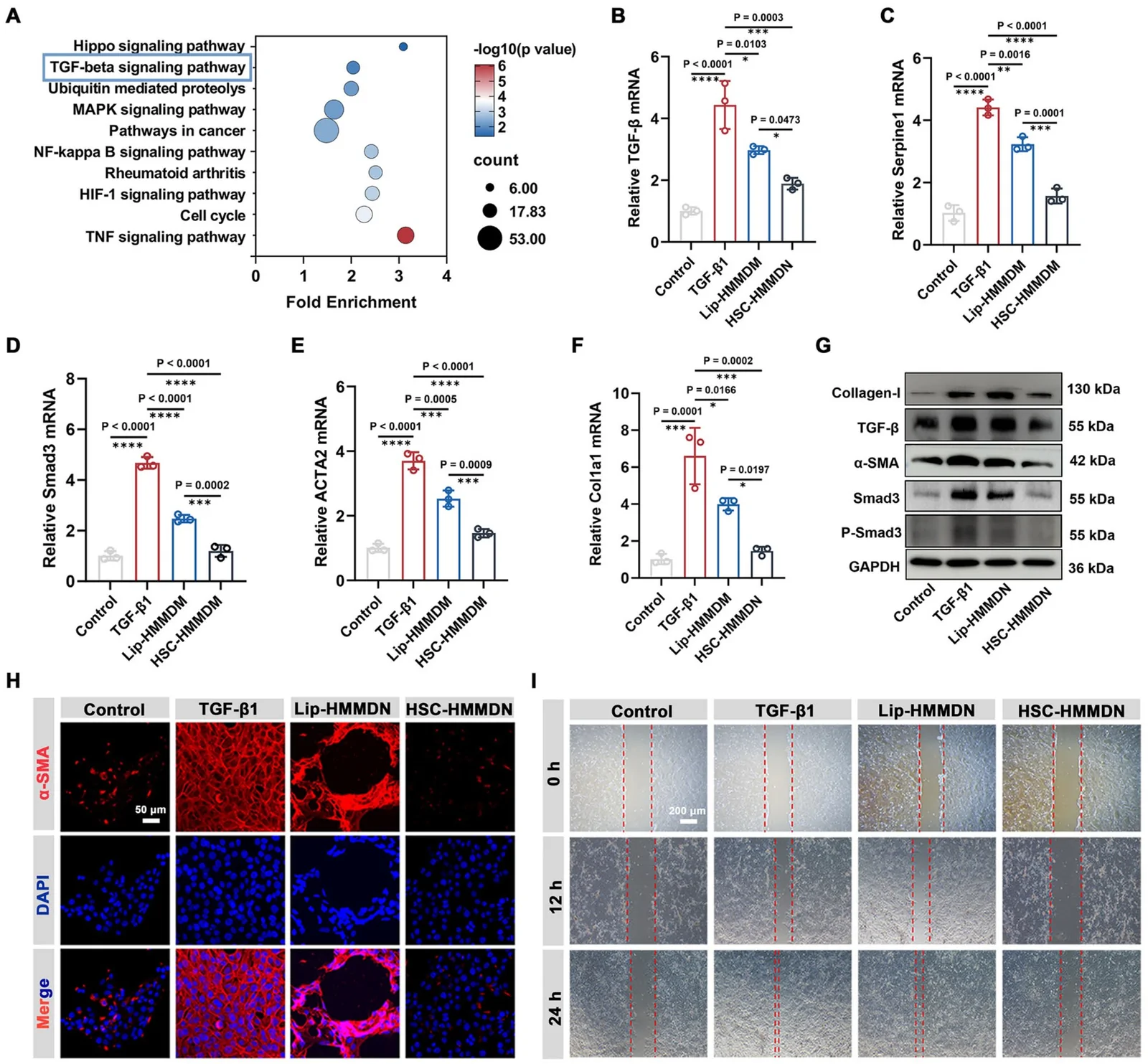
Effect of HSC-HMMDN on inhibiting HSC-T6 cells activation and migration. (**A**) KEGG pathway enrichment analysis of identified differentially expressed genes in the TGF-β1 and TGF-β1 + HSC-HMMDN groups. mRNA expressions of (**B**) TGF-β, (**C**) Serpine1, (**D**) Smad3, (**E**) ACTA2 and (**F**) Col1a1 in HSC-T6 cells. (**G**) Western blot assessment of collagen-I, TGF-β, α-SMA, Smad 3 and P-Smad3 in HSC-T6 cells. (**H**) Immunostaining of α-SMA of the HSC-T6 cells incubation with TGF-β1 and treated with Lip-HMMDN and HSC-HMMDN, respectively. The scale bar: 50 μm. (**I**) Images for cell scratch assay of HSC-T6 cells at 0, 12 and 24 h. Scale bar: 200 μm. Data represent mean ± SD (*n* = 3). Significance levels: *P < 0.05, **P < 0.01, ***P < 0.001, ****P < 0.0001.

To assess the impact of HSC-HMMDN on activated HSCs migration, we performed a scratch-wound assay. TGF-β1-stimulated activated HSCs achieved significantly faster wound closure within 24 h than that in unstimulated controls, indicative of increased proliferation and migration. In contrast, NPs treatment markedly inhibited HSC migration, as reflected by significantly wider scratch gaps compared to the model group. In particular, HSC-HMMDN exhibited the strongest effect, reducing wound closure to near-control levels (Figure 5I and Supplementary Figure S13). These results demonstrate that HSC‑HMMDN significantly inhibits the migration of TGF‑β1‑activated HSCs compared with Lip‑HMMDN, further supporting its superior antifibrotic efficacy.

### HSC-HMMDN improves liver function and alleviates liver fibrosis

The promising cellular therapeutic performance of HSC-HMMDN, along with their successful targeting in rats with liver fibrosis, prompted us to assess their *in vivo* anti‑fibrotic effects. The CCl_4_-induced rat model was selected for the assessment of HSC-HMMDN therapeutic potential for its ability to effectively replicate the fibrosis pathogenesis driven by oxidative stress from various sources, such as lifestyle factors, aging and liver injury [49, 50]. The detailed treatment protocol is shown in Figure 6A.

**Figure 6:**
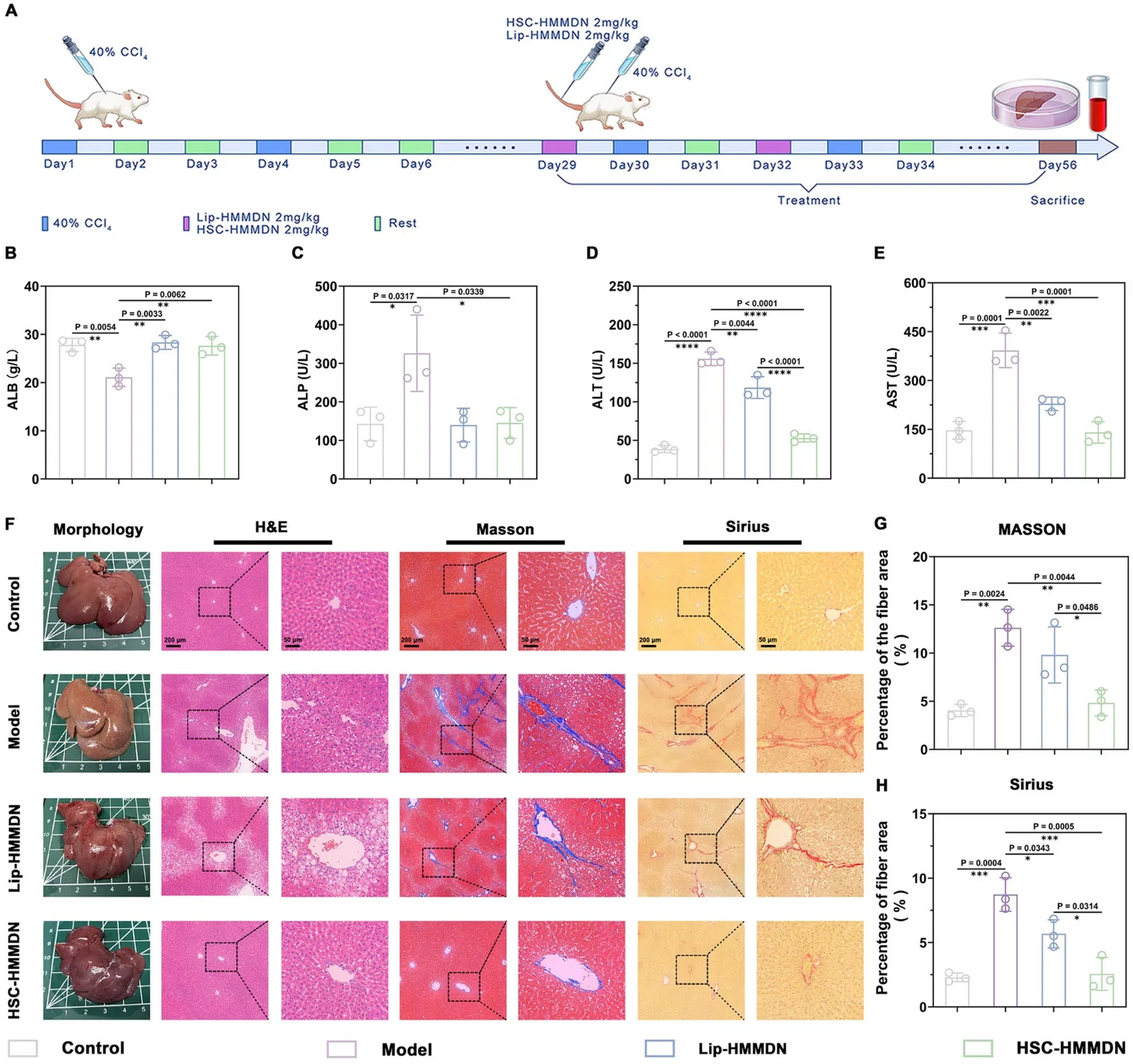
Efficacy of HSC-HMMDN on CCl_4_-induced liver fibrosis rats *in vivo*. (**A**) Schematic illustration of the treatment process for rat liver fibrosis. Serological levels of (**B**) ALB, (**C**) ALP, (**D**) ALT and (**E**) AST level. (**F**) Macroscopic examination and pathological changes in rat liver tissue following staining with H&E, Masson’s trichrome and Sirius red (scale bar = 200 μm and 50 μm). Quantitative analysis of collagen content in (**G**) Masson staining and (**H**) Sirius red staining. Data represent mean ± SD (*n* = 3). Significance levels: *P < 0.05, **P < 0.01, ***P < 0.001, ****P < 0.0001.

The progression of liver fibrosis is often associated with liver injury. In this study, eight weeks of CCl_4_ treatment significantly elevated serum ALP, AST and ALT levels while reducing ALB levels. Notably, treatment with Lip-HMMDN and HSC-HMMDN both ameliorated these abnormalities, with HSC-HMMDN exhibiting a significantly greater recovery (Figure 6B–E).

Gross morphological examination revealed distinct signs of fibrosis in livers from the model group, including a rough surface, mottled granular lesions and noticeable hardening (Figure 6F). By contrast, livers from HSC-HMMDN-treated rats exhibited a near-normal appearance with a smooth surface and soft texture, whereas only intermediate improvement was observed in the Lip-HMMDN group. Histological analysis by hematoxylin and eosin (H&E) staining corroborated these findings. Severe liver damage, characterized by inflammatory cell infiltration, disorganized hepatic cords, steatosis and perivenular fibrosis, was observed in the model group. Lip-HMMDN treatment resulted in marginal improvement, while HSC-HMMDN treatment markedly preserved liver parenchymal architecture. Furthermore, HSC-HMMDN treatment significantly improved collagen deposition compared to the model group (Figure 6G and H). Quantitative analysis confirmed that the collagen-positive area was decreased from approximately 8.7% ± 1.3% in the model group to <2.6% ± 1.3% in the HSC-HMMDN group, a level comparable to that in healthy controls (2.3% ± 0.3%) and significantly lower than that achieved with Lip-HMMDN (5.7% ± 1.1%). In line with these results, HSC-HMMDN significantly reduced the elevated levels of the activated HSC markers α-SMA and collagen I, as well as the hypoxia marker HIF-1α, in fibrotic liver tissues (Figure 7A–C and Supplementary Figure S14). Taken together, these findings demonstrate that HSC-HMMDN effectively ameliorate CCl_4_-induced liver injury, hypoxic microenvironment and fibrosis.

**Figure 7:**
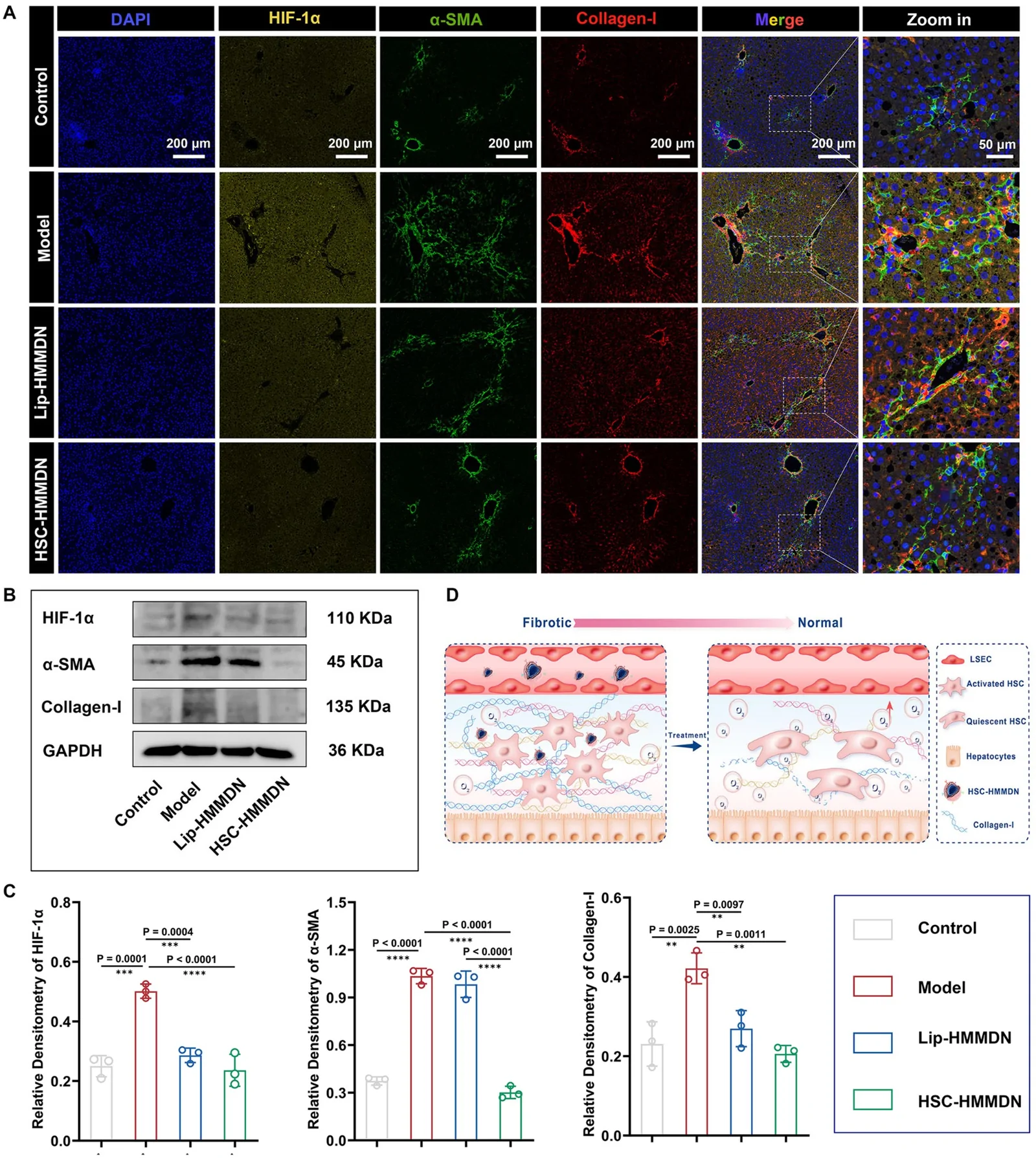
Analysis of protein expression in liver tissue. (**A**) Representative multiplex immunohistochemistry images of HIF-1α, α-SMA and collagen-I in the liver tissues. (**B**) Western blot assessment of HIF-1α, α-SMA and collagen-I in liver. (**C**) Quantitative analysis of the protein expression levels in (**B**). (**D**) Schematic illustration of the anti-fibrotic mechanism of HSC-HMMDN in fibrotic rats, including inhibiting HSCs activation, reducing collagen deposition and alleviating hypoxia. LSEC: liver sinusoidal endothelial cell. Data represent mean ± SD (*n* = 3). Significance levels: *P < 0.05, **P < 0.01, ***P < 0.001, ****P < 0.0001.

### Biosafety assessment

Assessing biological safety is a prerequisite for the clinical translation of novel nanomaterials. Initially, the cell viability of Lip-HMMDN and HSC-HMMDN was evaluated using CCK-8 assays. Even at 100 μg/mL, incubation with Lip-HMMDN and HSC-HMMDN resulted in viabilities exceeding 80% in BRL hepatocytes, indicating low cytotoxicity (Supplementary Figure S15).

Next, we mixed red blood cells with HSC-HMMDN at various concentrations. When nanomaterials are administered intravenously, their hemolysis rate must be kept under 5% as a critical safety requirement. Our results show that even at a concentration as high as 100 μg/mL, far exceeding the clinically relevant concentration expected from the *in vivo* dose in rats, the hemolysis rate of HSC-HMMDN remains below 5%, confirming its intravenous safety (Supplementary Figure S16).

Subsequently, serum levels of ALB, ALP, ALT and AST after HSC-HMMDN or Lip-HMMDN administration in healthy rats showed no significant change from controls and remained within the normal range, confirming minimal liver toxicity (Supplementary Figure S17). Then, major organs (heart, spleen, lung and kidneys) collected from the fibrotic rats in each group (treated with saline, Lip-HMMDN or HSC-HMMDN) were subjected to H&E staining and TUNEL staining. H&E staining revealed no noticeable histopathological abnormalities in these organs of rats after various treatments in the fibrosis model (Supplementary Figure S18). Additionally, TUNEL staining showed negligible green fluorescence in the treatment groups, demonstrating that Lip-HMMDN and HSC-HMMDN did not cause abnormal levels of cell death (Supplementary Figure S19). Collectively, these results demonstrate the favorable long-term biosafety of HSC-HMMDN and support its potential for clinical use.

## Conclusion

In this work, we developed a biomimetic nanozyme, HSC-HMMDN, for liver fibrosis therapy by coating HMMDN with activated HSCs-derived membranes. Leveraging homologous targeting mediated by integrins and N-cadherin, HSC-HMMDN achieves specific activated HSCs delivery. Its intrinsic SOD/CAT-mimetic activity orchestrates microenvironment remodeling by scavenging ROS and alleviating hypoxia. This dual action suppresses the downstream TGF-β/Smad pathway, effectively inhibiting HSC activation and alleviating liver fibrosis without apparent adverse effects. In summary, this microenvironment-remodeling strategy can open up new possibilities for anti-fibrotic treatment, holding promise for effective theranostics in the clinic.

## Supplementary Material

rbag163_Supplementary_Data

## Data Availability

Data will be made available on request.

## References

[1] Loomba R , Friedman SL, Shulman GI. Mechanisms and disease consequences of nonalcoholic fatty liver disease. Cell 2021;184:2537–64. 10.1016/j.cell.2021.04.015 PMC12168897 33989548

[2] Parola M , Pinzani M. Liver fibrosis in NAFLD/NASHsrom pathophysiology towards diagnostic and therapeutic strategies. Mol Aspects Med 2024;95:101231. 10.1016/j.mam.2023.101231 38056058

[3] Hu N , Guo C, Dai X, Wang C, Gong L, Yu L, Peng C, Li Y. Forsythiae fructuse water extract attenuates liver fibrosis via TLR4/MyD88/NF-κB and TGF-β/Smads signaling pathways. J Ethnopharmacol 2020;262:113275. 10.1016/j.jep.2020.113275 32810620

[4] Hernandez-Gea V , Friedman SL. Pathogenesis of liver fibrosis. Annu Rev Pathol 2011;6:425–56. 10.1146/annurev-pathol-011110-130246 21073339

[5] Vuppalanchi R , Noureddin M, Alkhouri N, Sanyal AJ. Therapeutic pipeline in nonalcoholic steatohepatitis. Nat Rev Gastroenterol Hepatol 2021;18:373–92. 10.1038/s41575-020-00408-y 33568794

[6] Friedman SL , Neuschwander-Tetri BA, Rinella M, Sanyal AJ. Mechanisms of NAFLD development and therapeutic strategies. Nat Med 2018;24:908–22. 10.1038/s41591-018-0104-9 PMC6553468 29967350

[7] Kisseleva T , Brenner D. Molecular and cellular mechanisms of liver fibrosis and its regression. Nat Rev Gastroenterol Hepatol 2021;18:151–66. 10.1038/s41575-020-00372-7 33128017

[8] Karsdal MA , Nielsen SH, Leeming DJ, Langholm LL, Nielsen MJ, Manon-Jensen T, Siebuhr A, Gudmann NS, Rønnow S, Sand JM, Daniels SJ, Mortensen JH, Schuppan D. The good and the bad collagens of fibrosis - their role in signaling and organ function. Adv Drug Deliv Rev 2017;121:43–56. 10.1016/j.addr.2017.07.014 28736303

[9] Higashi T , Friedman SL, Hoshida Y. Hepatic stellate cells as key target in liver fibrosis. Adv Drug Deliv Rev 2017;121:27–42. 10.1016/j.addr.2017.05.007 PMC5682243 28506744

[10] Tsuchida T , Friedman SL. Mechanisms of hepatic stellate cell activation. Nat Rev Gastroenterol Hepatol 2017;14:397–411. 10.1038/nrgastro.2017.38 28487545

[11] Xing L , Chang X, Shen L, Zhang C, Fan Y, Cho C, Zhang Z, Jiang H. Progress in drug delivery system for fibrosis therapy. Asian J Pharm Sci 2021;16:47–61. 10.1016/j.ajps.2020.06.005 PMC7878446 33613729

[12] Dewidar B , Meyer C, Dooley S, Meindl-Beinker AN. TGF-β in hepatic stellate cell activation and liver fibrogenesis - updated 2019. Cells 2019;8:1419. 10.3390/cells8111419 PMC6912224 31718044

[13] Jiang R , Zhou Y, Wang S, Pang N, Huang Y, Ye M, Wan T, Qiu Y, Pei L, Jiang X, Huang Y, Yang H, Ling W, Li X, Zhang Z, Yang L. Nicotinamide riboside protects against liver fibrosis induced by CCl_4_ via regulating the acetylation of Smads signaling pathway. Life Sci 2019;225:20–8. 10.1016/j.lfs.2019.03.064 30928408

[14] Xiang D , Zou J, Zhu X, Chen X, Luo J, Kong L, Zhang H. Physalin D attenuates hepatic stellate cell activation and liver fibrosis by blocking TGF-β/Smad and YAP signaling. Phytomedicine 2020;78:153294. 10.1016/j.phymed.2020.153294 32771890

[15] Jiao K , Yang K, Wang J, Ni Y, Hu C, liu J, Zhou M, Zheng J, Li Z. 27-Hydroxycholesterol induces liver fibrosis via down-regulation of trimethylation of histone H3 at lysine 27 by activating oxidative stress; effect of nutrient interventions. Free Radic Biol Med 2024;210:462–77. 10.1016/j.freeradbiomed.2023.11.043 38056577

[16] Peng Y , Lei H, Zhao J, Wang H, Luo Z, Wang D, Shi S, Wang T, Li J, Pang Z, Wang B, Xiong X. GDF10 attenuates MASH progression by restoring quiescent hepatic stellate cells via competitive inhibition of TGF-β/SMAD2 signaling. Int J Biol Sci 2025;21:6997–7012. 10.7150/ijbs.123784 PMC12631229 41281741

[17] Chen C , Chen J, Wang Y, Fang L, Guo C, Sang T, Peng H, Zhao Q, Chen S, Lin X, Wang X. Ganoderma lucidum polysaccharide inhibits HSC activation and liver fibrosis via targeting inflammation, apoptosis, cell cycle, and ECM-receptor interaction mediated by TGF-β/Smad signaling. Phytomedicine 2023;110:154626. 10.1016/j.phymed.2022.154626 36603342

[18] Tang Z , Li X, Tian L, Sun Y, Zhu X, Liu F. Mesoporous polydopamine based biominetic nanodrug ameliorates liver fibrosis via antioxidation and TGF-β/SMADS pathway. Int J Biol Macromol 2023;248:125906. 10.1016/j.ijbiomac.2023.125906 37482153

[19] Zhang J , Yao M, Xia S, Zeng F, Liu Q. Systematic and comprehensive insights into HIF-1 stabilization under normoxic conditions: implications for cellular adaptation and therapeutic strategies in cancer. Cell Mol Biol Lett 2025;30:2. 10.1186/s11658-024-00682-7 PMC11702238 39757165

[20] Zhang J , Zhang T, Zhang Z, Jia Q, Chen J, Li J, Song H, Li J, Xiong Y, Mo L, He J, Li Y. The dual‐target nanoparticles with ROS sensitivity inhibit the hedgehog signaling pathway and decrease oxidative stress in activated hepatic stellate cells to alleviate liver fibrosis. Adv Funct Materials 2024;34:2404592.

[21] Diebold I , Petry A, Hess J, Görlach A, Gruenberg J. The NADPH oxidase subunit NOX4 is a new target gene of the hypoxia-inducible factor-1. Mol Biol Cell 2010;21:2087–96. 10.1091/mbc.E09-12-1003 PMC2883952 20427574

[22] Choi J , Kim Y, Abeysiriwardhana HNI, Malla A, Kim JW, Anandappa JM, Liang Z, Seo S, Liu KH, Rhee S, Nahm SS, Kim ET, Lim Y, Cho M. Oxidative hypoxia drives TGF-β1-induced fibrosis under normoxia. Redox Biol 2026;89:103947. 10.1016/j.redox.2025.103947 PMC12721191 41344160

[23] Herranz-Itúrbide M , Peñuelas-Haro I, Espinosa-Sotelo R, Bertran E, Fabregat I. The TGF-β/NADPH oxidases axis in the regulation of liver cell biology in health and disease. Cells 2021;10:2312. 10.3390/cells10092312 PMC8470857 34571961

[24] Liang S , Kisseleva T, Brenner DA. The role of NADPH oxidases (NOXs) in liver fibrosis and the activation of myofibroblasts. Front Physiol 2016;7:17. 10.3389/fphys.2016.00017 PMC4735448 26869935

[25] Baghaei K , Mazhari S, Tokhanbigli S, Parsamanesh G, Alavifard H, Schaafsma D, Ghavami S. Therapeutic potential of targeting regulatory mechanisms of hepatic stellate cell activation in liver fibrosis. Drug Discov Today 2022;27:1044–61. 10.1016/j.drudis.2021.12.012 34952225

[26] Blas-García A , Apostolova N. Novel therapeutic approaches to liver fibrosis based on targeting oxidative stress. Antioxidants 2023;12:1567. 10.3390/antiox12081567 PMC10451738 37627562

[27] He H , Zhang Q, Zhang Y, Qu S, Li B, Lin J, Lu X, Xie C. Injectable bioadhesive and lubricating hydrogel with polyphenol mediated single atom nanozyme for rheumatoid arthritis therapy. Nat Commun 2025;16:2768. 10.1038/s41467-025-58059-z PMC11926226 40113809

[28] Padaga SG , Paul M, Banerjee T, Goswami S, Ghosh B, Biswas S. Hybrid metallic nanozyme with nitric oxide-releasing photothermal coating for accelerated infected diabetic wound healing. J Nanobiotechnology 2025;23:630. 10.1186/s12951-025-03693-4 PMC12512412 41068912

[29] Yao Y , Gao Z, Pan Z, Zhong Y, Qin L, Zhao K, Liu S, Zhang S, Han H, Yin F, Liang R, Zhao J, Zheng L. Ultrasound-triggered piezocatalytic MOF(Hf)-Pt nanozymes for efficient ROS scavenging in the treatment of osteoarthritis. Chem Eng J 2025;522:167229.

[30] Peng W , Tai W, Li B, Wang H, Wang T, Guo S, Zhang X, Dong P, Tian C, Feng S, Yang L, Cheng G, Zheng B. Inhalable nanocatalytic therapeutics for viral pneumonia. Nat Mater 2025;24:637–48. 10.1038/s41563-024-02041-5 39592721

[31] Wei T , Pan T, Peng X, Zhang M, Guo R, Guo Y, Mei X, Zhang Y, Qi J, Dong F, Han M, Kong F, Zou L, Li D, Zhi D, Wu W, Kong D, Zhang S, Zhang C. Janus liposozyme for the modulation of redox and immune homeostasis in infected diabetic wounds. Nat Nanotechnol 2024;19:1178–89. 10.1038/s41565-024-01660-y 38740936

[32] Mao Y , Gong Y, Bai X. The dual-faceted role of metal-Based nanomaterials in hepatic fibrosis therapy. Int J Nanomed 2026;21:574077. 10.2147/IJN.S574077 PMC13003978 41869402

[33] Ji H , Huo Y, Jiang L, Wen C, Guo E, Miao L, Li Y, Li T. A multifunctional GLP-MnO_2_ nanozyme suppresses TGF-β1/Smad signaling through ROS scavenging and dual-modality MRI/PAI-guided imaging for treating liver fibrosis. Biomater Adv 2026;184:214792. 10.1016/j.bioadv.2026.214792 41775176

[34] Xu L , Yang J, Cao X, Chen J, Liu Z, Cai L, Yu Y, Huang H. Sequential system based on ferritin delivery system and cell therapy for modulating the pathological microenvironment and promoting recovery. Int J Pharmaceut 2024;664:124607. 10.1016/j.ijpharm.2024.124607 39159856

[35] Truong Hoang Q , Nguyen Cao TG, Kang SJ, Lee M, Kang JH, Park HS, Kim JE, Bhang SH, Ko YT, Rhee WJ, Shim MS. Exosome membrane-sheathed and multi-stimuli-responsive MnO_2_ nanoparticles with self-oxygenation and energy depletion abilities potentiate the sonodynamic therapy of hypoxic tumors. Chem Eng J 2023;472:144871.

[36] Jiang X , Gray P, Patel M, Zheng J, Yin JJ. Crossover between anti- and pro-oxidant activities of different manganese oxide nanoparticles and their biological implications. J Mater Chem B 2020;8:1191–201. 10.1039/c9tb02524c 31967629

[37] Savchak OK , Wang N, Ramos-Docampo MA, de Dios Andres P, Sebastião AM, Ribeiro FF, Armada-Moreira A, Städler B, Vaz SH. Manganese dioxide nanosheet-containing reactors as antioxidant support for neuroblastoma cells. J Mater Chem B 2022;10:4672–83. 10.1039/d2tb00393g 35674248

[38] Yuan Y , Li J, Chen M, Zhao Y, Zhang B, Chen X, Zhao J, Liang H, Chen Q. Nano-encapsulation of drugs to target hepatic stellate cells: toward precision treatments of liver fibrosis. J Control Release 2024;376:318–36. 10.1016/j.jconrel.2024.10.012 39413846

[39] Kaps L , Limeres MJ, Schneider P, Svensson M, Zeyn Y, Fraude S, Cacicedo ML, Galle PR, Gehring S, Bros M. Liver cell type-specific targeting by nanoformulations for therapeutic applications. Int J Mol Sci 2023;24:17369. 10.3390/ijms241411869 PMC10380755 37511628

[40] Peng X , Yang L, Yuan P, Ding X. Hybrid cell membrane‐based nanoplatforms for enhanced immunotherapy against cancer and infectious diseases. Adv Healthc Mater 2024;13:e2304477. 10.1002/adhm.202304477 38709914

[41] Du Y , Ding H, Chen Y, Gao B, Mao Z, Wang W, Ding Y. A genetically engineered biomimetic nanodecoy for the treatment of liver fibrosis. Adv Sci 2024;11:e2405026. 10.1002/advs.202405026 PMC11516072 39206941

[42] Cheng Z , Li F, Qie Y, Sun J, Wang Y, Zhao Y, Nie G. Hepatic stellate cell membrane-camouflaged nanoparticles for targeted delivery of an antifibrotic agent to hepatic stellate cells with enhanced antifibrosis efficacy. Nano Lett 2024;24:15827–36. 10.1021/acs.nanolett.4c04820 39585320

[43] Fang RH , Gao W, Zhang L. Targeting drugs to tumours using cell membrane-coated nanoparticles. Nat Rev Clin Oncol 2023;20:33–48. 10.1038/s41571-022-00699-x 36307534

[44] Stöber W , Fink A, Bohn E. Controlled growth of monodisperse silica spheres in the micron size range. J Colloid Interface Sci 1968;26:62–9.

[45] Shao M , Wang Y, Dong H, Wang L, Zhang X, Han X, Sang X, Bao Y, Peng M, Cao G. From liver fibrosis to hepatocarcinogenesis: role of excessive liver H_2_O_2_ and targeting nanotherapeutics. Bioact Mater 2023;23:187–205. 10.1016/j.bioactmat.2022.11.001 PMC9663332 36406254

[46] Islam MN , Rauf A, Fahad FI, Emran TB, Mitra S, Olatunde A, Shariati MA, Rebezov M, Rengasamy KRR, Mubarak MS. Superoxide dismutase: an updated review on its health benefits and industrial applications. Crit Rev Food Sci Nutr 2022;62:7282–300. 10.1080/10408398.2021.1913400 33905274

[47] Hintermann E , Christen U. The many roles of cell adhesion molecules in hepatic fibrosis. Cells 2019;8:1503. 10.3390/cells8121503 PMC6952767 31771248

[48] Nolfi-Donegan D , Braganza A, Shiva S. Mitochondrial electron transport chain: oxidative phosphorylation, oxidant production, and methods of measurement. Redox Biol 2020;37:101674. 10.1016/j.redox.2020.101674 PMC7767752 32811789

[49] Dong S , Chen QL, Song YN, Sun Y, Wei B, Li XY, Hu YY, Liu P, Su SB. Mechanisms of CCl_4_-induced liver fibrosis with combined transcriptomic and proteomic analysis. J Toxicol Sci 2016;41:561–72. 10.2131/jts.41.561 27452039

[50] Hayashi S , Itoh A, Isoda K, Kondoh M, Kawase M, Yagi K. Fucoidan partly prevents CCl_4_-induced liver fibrosis. Eur J Pharmacol 2008;580:380–4. 10.1016/j.ejphar.2007.11.015 PMC2258315 18068155

