# Supplementary material for: Biomimetic dual-catalytic nanozyme remodels the liver fibrotic microenvironment to alleviate fibrosis
Source: Regen Biomater. 2026 Nov 3;13:rbag163. doi: 10.1093/rb/rbag163 (PMC13742282; doi:10.1093/rb/rbag163)
Supplement: Supplementary file 1 [file rbag163_supplementary_data.zip › Table S1-S2.docx]

**Table S1. Antibodies used**

| Antibody | Catalog | Company | Application | Dilution |
| --- | --- | --- | --- | --- |
| N-cadherin antibody | BYab-17052 | BYabscience (Nanjing, China). | IF | 1:1000 |
| E-cadherin antibody | BYab-17017 | BYabscience (Nanjing, China). | IF | 1:1000 |
| Integrin αv | BYab-17098 | BYabscience (Nanjing, China). | IF | 1:1000 |
| Integrin β3 | BYab-17033 | BYabscience (Nanjing, China). | IF | 1: 1000 |
| Integrin β1 | BYab-17031 | BYabscience (Nanjing, China). | IF | 1: 1000 |
| α-SMA | ET1607-53 | HUABIO (Hangzhou, China) | WB/IF | 1:5000 |
| α-SMA | ab150301 | Abcam (Cambridge, UK) | mIHC | 1: 1000 |
| Collagen-I | ab270993 | Abcam (Cambridge, UK) | mIHC/WB | 1: 1000 |
| HIF-1α | MAB1536 | R&D (Minneapolis, MN, USA) | mIHC/WB | 1:1000 |
| GAPDH | ET1601-4 | HUABIO (Hangzhou, China) | WB | 1:20000 |
| TGF-β | HA723604 | HUABIO (Hangzhou, China) | WB | 1:2000 |
| Smad3 | ET1607-41 | HUABIO (Hangzhou, China) | WB | 1:5000 |
| p-Smad | HA750172 | HUABIO (Hangzhou, China) | WB | 1:2000 |
| Tom20 | 42406 | Cell Signaling Technology (Danvers, MA, USA) | IF | 1:1000 |
| iFluor™ 594 Conjugated Goat anti-rabbit IgG polyclonal Antibody | HA1122 | HUABIO (Hangzhou, China) | IF | 1:2000 |
| iFluor™ 488 Conjugated Goat anti-rabbit IgG polyclonal Antibody | HA1121 | HUABIO (Hangzhou, China) | IF | 1:2000 |

**Table S2. Primer sequences for RT-PCR.**

| **Primer** | **Forward** | **Reverse** |
| --- | --- | --- |
| 01 | TGFβ-F | ACGTGGAGCTGTACCAGAAA |
| 02 | TGFβ-R | GCAGTGTGTTATCCCTGCTG |
| 03 | Smad3-F | AAGATCCCACCAGGATGCAA |
| 04 | Smad3-R | GGTCAACTGGTAGACAGCCT |
| 05 | Col1a1-F | CACCAATCACCTGCGTACAG |
| 06 | Col1a1-R | GCAGTTCTTGGTCTCGTCAC |
| 07 | ACTA2-F | GGTGCTGTCTCTCTATGCCT |
| 08 | ACTA2-R | AAGGAATAGCCACGCTCAGT |
| 09 | Serpine1-F | CCGCCTCTTCCACAAATCAG |
| 10 | Serpine1-R | GTAGGGCAGTTCCAGGATGT |
| 11 | ACTIN-F | CACTCTTCCAGCCTTCCTTC |
| 12 | ACTIN-R | GTACAGGTCTTTGCGGATGT |
