# Supplementary material for: Biomimetic dual-catalytic nanozyme remodels the liver fibrotic microenvironment to alleviate fibrosis
Source: Regen Biomater. 2026 Nov 3;13:rbag163. doi: 10.1093/rb/rbag163 (PMC13742282; doi:10.1093/rb/rbag163)
Supplement: Supplementary file 1 [file rbag163_supplementary_data.zip › Supplement_.docx]

**Figure S1.** The hydrodynamic diameter distribution of HSC-HMMDN NPs.

**Figure S2.** Stability assessment of HSC-HMMDN NPs over 7 days showing DLS size (A) and Zeta potential (B) measurements. Data represent mean ± SD (n = 3).

**Figure S3.** N_2_ adsorption-desorption isotherms, and pore size distributions of HMMDN

**Figure S4.** XRD image of HMMDN.

**Figure S5.** Determination of the dissolved oxygen.

**Figure S6.** Quantitative analysis of cellular uptake at 12 h and 24 h. Data represent mean ± SD (n = 3). Significance levels: *****p* < 0.0001.

**Figure S7.** Quantitative analysis of fluorescence images in CCl_4_-induced liver fibrotic rats after injection with HSC-HMMDN NPs and Lip-HMMDN NPs at various time points. Data represent mean ± SD (n = 3).

**Figure S8.** Quantitative analysis of fluorescence images for major organs after post-injection for 24 h. Data represent mean ± SD (n = 3).

**Figure S9.** Quantitative ROS production in HSC-T6 cells treated with the various treatments, respectively. Data represent mean ± SD (n = 3). Significance levels: *****p* < 0.0001.

**Figure S10.** (D) Quantification of red fluorescent J-aggregates and green monomers in HSC-T6 cells treated with various treatments, respectively. Data represent mean ± SD (n = 3). Significance levels: *****p* < 0.0001.

**Figure S11.** Relative protein expression levels for Collagen-I (A), α-SMA (B), TGF-β (C), Smad3 (D) and P-Smad3 (E). Data represent mean ± SD (n = 3). Significance levels: **p* < 0.05, ***p* < 0.01, ****p* < 0.001, *****p* < 0.0001.

**Figure S12.** Quantitative fluorescence analysis of α-SMA in HSCs treated with different treatments. Data represent mean ± SD (n = 3). Significance levels: **p* < 0.05, *****p* < 0.0001.

**Figure S13.** Quantitative statistics of the degree of migration of HSC-T6 cells. Data represent mean ± SD (n = 3). Significance levels: ***p* < 0.01, ****p* < 0.001, *****p* < 0.0001.

**Figure S14.** Quantitative analysis of fluorescence images of HIF-1α (A), α-SMA (B), and Collagen I (C) in rats from different treatment groups. Data represent mean ± SD (n = 3). Significance levels: **p* < 0.05, ***p* < 0.01, ****p* < 0.001, *****p* < 0.0001.

**Figure S15.** Cytotoxicity of various concentrations of HSC-HMMDN NPs (A) and Lip-HMMDN NPs (B) to BRL cells. Data are presented as mean ± SD (n = 3).

**Figure S16.** Hemolysis test of HSC-HMMDN NPs with different concentrations. Data represent mean ± SD (n = 3).

**Figure S17.** Serum alanine aminotransferase (ALT), alkaline phosphatase (ALP), aspartate aminotransferase (AST), and albumin (ALB) levels in healthy rats after treatment with HMMDN and HSC-HMMDN. Data represent mean ± SD (n = 3).

**Figure S18.** Representative H&E staining images showing the tissue structures of heart, spleen, lungs, and kidneys of fibrotic rat treated with Lip-HMMDN NPs and HSC-HMMDN NPs. Scale bars: 100 μm.

**Figure S19.** Representative TUNEL staining images showing the apoptosis cells of heart, spleen, lungs, and kidneys of fibrotic rat treated with Lip-HMMDN and HSC-HMMDN. Scale bars: 50 μm.
